# Identifying vital nodes for influence maximization in attributed networks

**DOI:** 10.1038/s41598-022-27145-3

**Published:** 2022-12-31

**Authors:** Ying Wang, Yunan Zheng, Yiguang Liu

**Affiliations:** College of Computer Science, Sichuan University, Chengdu, 610065 Sichuan China

**Keywords:** Computational science, Information technology

## Abstract

Identifying a set of vital nodes to achieve influence maximization is a topic of general interest in network science. Many algorithms have been proposed to solve the influence maximization problem in complex networks. Most of them just use topology information of networks to measure the node influence. However, the node attribute is also an important factor for measuring node influence in attributed networks. To tackle this problem, we first propose an extension model of linear threshold (LT) propagation model to simulate the information propagation in attributed networks. Then, we propose a novel community-based method to identify a set of vital nodes for influence maximization in attributed networks. The proposed method considers both topology influence and attribute influence of nodes, which is more suitable for identifying vital nodes in attributed networks. A series of experiments are carried out on five real world networks and a large scale synthetic network. Compared with CELF, IMM, CoFIM, HGD, NCVoteRank and K-Shell methods, experimental results based on different propagation models show that the proposed method improves the influence spread by $-2.28\% \, \textrm{to} \, 4.76\%$, $-2.50\% \, \textrm{to} \, 16.97\%$, $0.18\% \, \textrm{to} \, 16.07\%$, $0.22\% \, \textrm{to} \, 41.82\%$, $0.23\% \, \textrm{to} \, 11.24\%$ and $10.78\% \, \textrm{to} \, 75.22\%$.

## Introduction

Complex networks are common in real world and can be used to represent complex systems in many fields. More and more complex networks come with attributes in nodes and are named as attributed networks^1^. These networks not only contain topology structures, but also have rich node attribute information such as text descriptions of nodes and comments related to nodes. Influence maximization (IM) is a classic optimization problem in network science, which aims to seek a set of vital nodes that the diffusion orients from these nodes can cause the maximum influence spread in networks. Vital nodes identification for IM has been widely used in many applications such as viral marketing^2^, information propagation^3^, rumor analysis^4^ and so on.

Many IM algorithms have been proposed in complex networks, including diffusion-based algorithms^5–7^ and heuristic-based algorithms^8–12^. Diffusion-based algorithms provide a good performance guarantee to the optimal solution with the weakness of enormous calculations. Heuristic-based methods improve efficiency to some extent but take no consideration of propagation models or do not optimize a global function of influence. Recently, community-based methods^13–15^ play an important role in the IM problem. A community is defined as a group of nodes with dense internal connections and relatively sparse connections to the rest of the network. It can effectively represents the organization and structure of the network^16^. Benefiting from the fact that different communities are sparsely connected, the propagation overlap between seed nodes selected from different communities can be effectively reduced.

Due to the benefits of community-based influence maximization algorithms, many previous studies have focused on them in complex networks. The first and foremost step of community-based algorithms is community detection. Numerous community detection methods based on matrix factorization^17,18^, label propagation^19,20^, percolation^21^ and random walks^22,23^ have been proposed with certain limitations and scalability issues. However, these community detection methods only use the information relevant to the graph topology and fail to correlate node features with the community structure^24^. Recently, the graph-embedding based community detection methods^25,26^ have attracted tremendous attention, since they can learn a representation that embeds the topology into the attribute for each node. Given the good performance of graph-embedding methods in community detection, we try to apply it to solve the influence maximization problem.

Although many community-based methods have been proposed for the IM problem, there are few methods that are suitable for attribute networks. Almost all graph clustering or community detection methods in attribute networks do not conduct the influence maximization study since there are no suitable information propagation models for attributed networks. Moreover, community-based influence maximization algorithms avoid the propagation overlap between seed nodes selected from different communities, but the propagation overlap between seed nodes selected from the same community may still exists which may reduce the influence spread. To solve the above problems, we propose an information propagation model and a novel community-based influence maximization algorithm for attributed networks. The main contributions are summarized as following:

- An extension of classic linear threshold (LT) information propagation model is proposed named LTPlus, which not only considers topology structures of networks but also attributes of nodes.
- To solve the influence maximization problem in attributed networks, we propose a community-based influence maximization algorithm using graph-embedding. To the best of our knowledge, it is the first time that a graph-embedding based community detection method is used to the influence maximization problem.
- The proposed method alleviates the propagation overlap between seed nodes selected from the same community by recalculating the influence of seed nodes’ predecessors during the seed nodes selection process.
- Extensive analysis is performed on six datasets, and experimental results show that the proposed method has a good performance.

## Related work

The related IM algorithms in this paper are classified into three categories: diffusion-based methods, heuristic-based methods and community-based methods. These methods are discussed with more details below:

Kempe et al.^5^ proposed the diffusion-based method, Greedy, which provides a $(1-1/e-\varepsilon )$ approximation performance guarantee to the optimal solution. However, its computation cost is expensive since it needs to perform Monte-Carlo simulations on all possible combinations of the current seed set and remaining nodes. Leskovec et al.^6^ proposed the CELF algorithm which employed the principle of diminishing marginal utility to avoid a lot of Monte-Carlo simulations. It significantly reduces the time complexity but it is still not scalable to large scale networks.

To improve efficiency, some heuristic centrality measures, such as degree centrality^27^, K-Shell^9^, betweenness centrality^28^ and closeness centrality^29^ etc., were proposed to evaluate node influence. Moreover, Li et al.^3,30^ proposed to identify influential nodes by novel gravity models. LENC^12^ identified influential nodes by the entropy of the node based on the weight distribution of edges connected to it. However, these methods may lead to rich-club effect in solving the IM problem. VoteRank^31^ was proposed to reduce the rich-club effect by selecting seed nodes based on a voting scheme, where the voting ability of each node is the same and each node gets the vote from its neighbors. NCVoteRank^32^ argued that the voting ability of each node should be different and depends on its topological position. A fast and accurate IM algorithm, LMP^33^, was proposed by using a local traveling for labeling of nodes based on the influence power. This method can achieve a linear time complexity, while have good performance. HGD^34^ presented a heuristic group discovery method to reduce the influence overlap, which utilized the K-Shell and degree centrality to cluster nodes. However, HGD is a local optimal clustering algorithm that cannot guarantee global optimal performance. Overall, heuristic-based methods are relatively time efficiency but may lack performance guarantee in some networks.

As the community detection is an appropriate approach for understanding the structure and hidden information in complex networks^35^, many community-based IM methods were proposed. Li et al.^36^ pointed out that higher community diversity can reduce the risk of marketing campaigns and prolong the effect of a marketing campaign in the future promotion. OASNET^37^ used the Clauset-Newman-Moore community detection method and selected candidate nodes from each community by classic greedy-based algorithm, then selected seed nodes from candidates by dynamic programming. However, the efficiency of this method still need to be improved. A fast overlapping community-based IM method, FIP^33^, was proposed by removing insignificant communities to decrease the search space for choosing seed nodes. This makes the method time efficient. The probability coefficient of global diffusion is considered to improve seed node selection performance. CoFIM^38^ used the Louvain algorithm^39^ for community detection and defined the node-expansion and intra-community propagation under the weighted cascade model, which successfully avoid thousand times of Monte-Carlo simulations. This method performs well on many large-scale datasets and has high time efficiency.

However, these aforementioned methods just focus on network topologies and fail to measure the importance of node attributes in attributed networks, while the attribute is also an essential indicator as well as the topology. Some literature^40,41^ dealt with node attributes and studied target-aware IM problem, but their optimization objective functions are different from traditional IM. Besides, the continued growth of the network scale and high-dimensional node attributes put forward higher requirements for the efficiency and scalability of community detection algorithms in attributed networks. Inspired by the significant progresses in graph-embedding^42^, graph-embedding based community detection came into view in recent years. AANE^43^ computed the attribute similarity matrix between nodes and calculated vector representation associated with structural information and designed the joint learning process in a distributed manner. He et al.^44^ cast MRFasGCN as an encoder for unsupervised community detection in attributed networks. AGC^45^, an adaptive graph convolution method, exploited high-order graph convolution to capture global cluster structure and adaptively selected the appropriate order for different networks. These graph-embedding methods only complete the community detection task, but do not solve the IM problem. Therefore, vital nodes identification for IM in attributed networks is still a challenging problem to be solved.

## Preliminaries

### Attributed networks

Given a directed and attributed network $G=(V,E,X)$, where $V=\{v_1,v_2,\ldots ,v_N\}$ is the set of nodes and $|V|=N$. *E* is the set of edges which can be represented as an adjacency matrix $A=\{a_{ij}\}\in {\mathbb {R}}^{N\times N}$, where $a_{ij}=1$ if node $v_{i}$ connects to node $v_{j}$ and otherwise $a_{ij}=0$. $X=[x_1,x_2,\ldots ,x_N]^{T}$ is the attribute matrix of all nodes, where $x_i\in {\mathbb {R}}^d$ is a real-valued attribute vector of node $v_i$ and *d* is the dimension of attribute.

### Linear threshold (LT) model

The LT model^5^ is a widely used information diffusion model. In the LT model, nodes are divided into two states: active and inactive. In a directed network, the activation of node $v_i$ depends on its in-neighbors $N_{in}(v_i)$. If $v_j\in N_{in}(v_i)$ is active, it has an influence on $v_i$, denoted as $b_{v_j,v_i}$. In the LT model, $b_{v_j,v_i}$ is set as:

1 $$\begin{aligned} b_{v_j,v_i} = \frac{1}{k_{in}(v_i)} , \end{aligned}$$

where $k_{in}(v_i)$ represents the in-degree of node $v_i$. Each node in $N_{in}(v_i)$ has an influence value to $v_i$, and the summation of these values must be no more than 1, that is $\sum _{v_j\in N_{in}(v_i)}b_{v_j,v_i}\le 1$. Each node $v_i$ has an activation threshold $\theta _{v_i}$ which is between 0 and 1. Therefore, $v_i$ will be activated once $\sum _{v_j\in N_{in}(v_i)}b_{v_j,v_i}\ge \theta _{v_i}$. The diffusion process is over until no more nodes can be activated.

### Independent cascade (IC) model

Another well-known information diffusion model is the IC model^46^. In the IC model, each edge has a probability *p* to measure the social influence of this edge. Nodes are also divided into active and inactive states. If a node $v_i$ is activated, then it has a chance with probability *p* to activate its inactive out-neighbor $v_j$ in a directed network.

### Influence maximization

Influence maximization^47^ aims to find a node subset $S\subseteq V$ and $|S|=m$, such that the expected influence scope is maximal:

2 $$\begin{aligned} S^* = \arg _S\max \phi (S), \end{aligned}$$

where $\phi (S)$ is an objective function used to evaluate the expected number of active nodes after the diffusion process.

### Well-known state-of-the-art methods

Four state-of-the-art IM methods are introduced in this paper. These algorithms have been proved^48,49^ to perform well on many datasets.

- *CELF*^6^: a much faster greedy-based algorithm based on the submodularity of the spread function. By using the principle of diminishing marginal utility, CELF achieves an up to 700 times improvement in running time while maintains similar practical performance compared with the simple greedy-based algorithm. However, the running time of CELF is still terrible especially on large-scale datasets which makes it meaningless in practical applications. Thus, we do not compare it on the Synthetic dataset in this paper.
- *IMM*^50^: a martingale-based algorithm which utilizes reverse influence sets^51^. It computes a lower bound of the maximum expected spread of *m* nodes and derives the number of random Reverse Reachable(RR) sets needed to be sampled. The first *m* nodes that appear most frequently in the RR sets are selected as seeds.
- *CoFIM*^38^: a community-based framework for influence maximization assuming that influence propagates from seed nodes to their neighbors and then from these neighbors to other nodes within the same community. Based on this assumption, an incremental greedy algorithm is developed to select seed nodes. In contrast to other community-based algorithms, CoFIM has high time efficiency.
- *HGD*^34^: a heuristic group discovery algorithm using centrality metrics and the strong community rule to cluster cohesive nodes into one group. Compared with other heuristic-based algorithms, HGD is more efficient and perform well especially when *m* is small since it is a local optimal algorithm.
- *NCVoteRank*^32^: a neighborhood coreness based voting approach designed to find spreaders by taking the coreness value of neighbors into consideration for the voting of node influence. NCVoteRank is also a heuristic-based algorithm, which outperforms many existing popular algorithms and is competitive in time complexity.
- *K-Shell*^9^: in this method, nodes that locate within the core of the network are identified to be more important by the K-Shell decomposition analysis. The top *k* nodes with larger K-Shell value are selected as seeds.

## Methods

### The proposed LTPlus propagation model

For a given directed and attributed network *G*, the LTPlus model considers both the topology influence and the attribute influence between nodes. In order to better compare with the LT model, we do not change the topology influence evaluation method in the classical LT model. Thus, the incoming topology influence of $v_i$ is the same as Eq. (1), and here it is noted as $TI_{in}(v_j,v_i)$:

3 $$\begin{aligned} TI_{in}(v_j,v_i)=\frac{1}{k_{in}(v_i)}, \end{aligned}$$

where $v_j$ is the in-neighbour of $v_i$.

Since node attributes represent common characteristics among nodes which play essential roles in the information diffusion, the incoming influence from in-neighbors in the LTPlus model is jointly decided by both the incoming topology influence and the incoming attribute influence. Similar attribute vectors mean that these nodes are homogenous, and the information propagation between these nodes will be easier. That is to say, the attribute influence will be greater if attribute vectors of two nodes are similar. We simply use the cosine similarity^52^ to measure the similarity of attribute vectors:

4 $$\begin{aligned} s_a(v_j,v_i) = \frac{x_i\cdot x_j}{\Vert x_i\Vert \cdot \Vert x_j\Vert }. \end{aligned}$$

In order to make the topology influence and attribute influence in the same order of magnitude, we adopt the edge-softmax^53^ method to normalize $s_a(v_j,v_i)$ for each node and get the incoming attribute influence of $v_i$:

5 $$\begin{aligned} AI_{in}(v_j,v_i) = \frac{s_a(v_j,v_i)}{\sum _{v_l \in N_{in}(v_i)} s_a(v_l,v_i)}, \end{aligned}$$

where $v_j$ is the in-neighbour of $v_i$, and $N_{in}(v_i)$ represents the in-neighbors set of $v_i$.

To sum up, the incoming influence of node $v_i$ from its in-neighbour $v_j$ is calculated as the linear combination of the incoming topology influence $TI_{in}(v_j,v_i)$ and the incoming attribute influence $AI_{in}(v_j,v_i)$. Thus, the incoming influence ${\hat{b}}_{v_j,v_i}$ in LTPlus model is defined as:

6 $$\begin{aligned} {\hat{b}}_{v_j,v_i} = \alpha _1 \cdot TI_{in}(v_j,v_i) + \alpha _2 \cdot AI_{in}(v_j,v_i), \end{aligned}$$

where $\alpha _1$ and $\alpha _2$ indicate the weight coefficients of topology and attribute influence, $\alpha _1, \alpha _2 \in (0,1)$ and $\alpha _1 + \alpha _2 = 1$.

Obviously, the LTPlus propagation model takes into account topology structure and attribute similarity between nodes. Besides, the LTPlus propagation model fully considers that different in-neighbors contribute different attribute influence, which is more in line with real situations of information propagation. When $\alpha _1 = 1$, the LTPlus model degenerate into the LT model, while $\alpha _1 = 0$ means only node attributes are considered in information diffusion process. Generally, we treat the topology and attribute influence on an equal basis and set $\alpha _1 = \alpha _2 = 0.5$.

#### The graph-embedding based community detection method

The goal of graph-embedding based community detection is to partition nodes in the network *G* into *l* clusters $C=\{C_1,C_2,\ldots ,C_l\}$. As mentioned above, an adaptive graph convolution (AGC) method^45^ is used in this paper as the community detection method. A low-pass graph filter *F*^45^ is designed in AGC:

7 $$\begin{aligned} F = I - \frac{1}{2}L_s, \end{aligned}$$

where $L_s = I-D^{-\frac{1}{2}}AD^{-\frac{1}{2}}$ is the symmetrically normalized graph Laplacian operator, *I* is the identity matrix and *D* is the degree matrix. To capture global graph structures and facilitate clustering, AGC defined *k*-order graph convolution^45^ as:

8 $$\begin{aligned} {\bar{X}}=(I-\frac{1}{2}L_s)^k X, \end{aligned}$$

where *k* is a positive integer. After convolution, AGC employed the linear kernel $K={\bar{X}}{\bar{X}}^T$ to learn pairwise similarity between nodes and then performed spectral clustering on $W=\frac{1}{2}(|K|+|K^T|)$ to obtain clustering results.

*k*-order graph convolution will produce smoother attributes as *k* increases, but too large *k* may lead to over-smoothing, i.e., the attributes of nodes in different clusters are mixed and become indistinguishable. To adaptively select the order *k*, the intra-cluster distance *intra*(*C*)^45^ is computed to measure clustering performance:

9 $$\begin{aligned} intra(C)= \frac{1}{|C|}\sum _{C_i\in C} \frac{1}{|C_i|(|C_i|-1)}\sum _{v_i,v_j\in C_i,v_i\ne v_j}\Vert \bar{x_i}-\bar{x_j}\Vert , \end{aligned}$$

where |*C*| is the number of communities and $|C_i|$ is the number of nodes in community $C_i$. This graph convolution network is trained iteratively until *intra*(*C*) converges.

However, AGC is designed for undirected networks. The symmetric operator $L_s$ cannot be directly used for directed networks, since adjacency matrices of directed networks are asymmetric. A simple but effective method is to construct a symmetric matrix $A_s$^54^:

10 $$\begin{aligned} A_s=A+A^T. \end{aligned}$$

Then, a degree matrix $D_s$ is built from $A_s$ and the Laplacian operator is $L_{sd} = I-D_s^{-\frac{1}{2}}A_sD_s^{-\frac{1}{2}}$. That is, the graph Laplacian operator $L_s$ in AGC is replaced by $L_{sd}$ in this paper. For the convenience of notation, the improved AGC method applicable for directed networks is noted as DAGC.

#### The seed nodes selection method

After community detection, nodes with powerful influence will be selected from different communities by measuring both topology and attribute influence. There are two key issues in the seed nodes selection phase: (1) The first problem is that how many nodes should be selected from each community. (2) The second problem is that how to select seed nodes.

To address the first problem, we empirically find that communities of different sizes should not be treated the same, since placing seed nodes in a large community could trigger more nodes than in a small community. According to this, a quota-based approach is adopted and $m_{C_i}$ nodes are selected from each community:

11 $$\begin{aligned} m_{C_i} = round(m \times \frac{|C_i|}{N}), \end{aligned}$$

where *round*() function means rounding the value to the nearest integer, and *m* is the total number of seed nodes. Thus, $m_{C_i}$ nodes will be selected from community $C_i$ and added to the seed node sequence. If the seed node sequence length is larger than or equal to *m*, the iteration will be broken. In contrast, if the seed node sequence is smaller than *m*, the node with the maximum influence in the current network will be selected as the seed node.
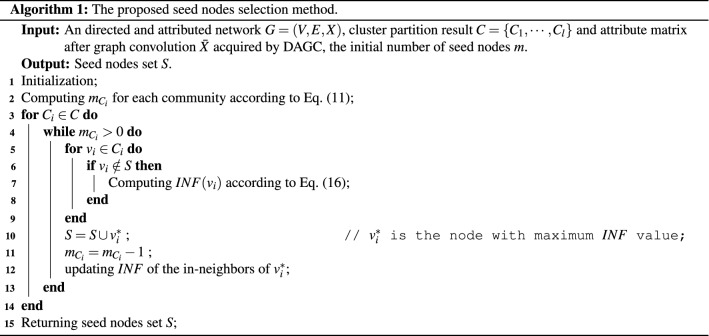


For the second key problem, when selecting influential nodes in directed networks, we pay more attention to how many nodes can be affected by one node. The more nodes it points to, the more nodes it can affect. Thus, the out-degree of each node is used to measure its topology influence, which can be formulated by:

12 $$\begin{aligned} TI_{out}(v_i)=k_{out}(v_i). \end{aligned}$$

The more similar the attributes between nodes, the more likely the information successfully propagates between these nodes. Thus, the attribute influence of a node is measured by its attribute similarities to its out-neighbors. Attributes after graph convolution ${\bar{X}}$ are used to compute cosine similarities for nodes since they integrates topology and attributes well. It is noteworthy that different from Eq. (4), the attribute similarity after convolution noted as $\overline{s_a}(v_i,v_k)$ is calculated between node $v_i$ and its out-neighbor $v_k$:

13 $$\begin{aligned} \overline{s_a}(v_i,v_k) = \frac{\bar{x_i}\cdot \bar{x_k}}{\Vert \bar{x_i}\Vert \cdot \Vert \bar{x_k}\Vert }. \end{aligned}$$

The attribute influence of a node is calculated by summing the attribute similarities to its all out-neighbors:

14 $$\begin{aligned} AI_{out}(v_i) = \sum _{v_k \in N_{out}(v_i)} \overline{s_a}(v_i,v_k), \end{aligned}$$

where $N_{out}(v_i)$ is the out-neighbors set of node $v_i$.

To ensure that the influence of each node is in the range of [0, 1], the topology and attribute influence of each node are normalized by Min-Max scaling normalization method. The normalization of $TI_{out}(v_i)$ and $AI_{out}(v_i)$ noted as $NTI(v_i)$ and $NAI(v_i)$ respectively are calculated as follows:

15 $$\begin{aligned} \left\{ \begin{aligned} NTI(v_i)= & {} \frac{TI_{out}(v_i)-min(TI_{out})}{max(TI_{out})-min(TI_{out})} \\ NAI(v_i)= & {} \frac{AI_{out}(v_i)-min(AI_{out})}{max(AI_{out})-min(AI_{out})}, \end{aligned} \right. \end{aligned}$$

where $max(TI_{out})$ and $min(TI_{out})$ are the maximal and minimal value of nodes’ topology influence respectively, and similarly $max(AI_{out})$ and $min(AI_{out})$ are the maximal and minimal value of nodes’ attribute influence respectively. The topology influence and the attribute influence are supposed to be treated on an equal basis. Thus, the total outcoming influence of each node is:

16 $$\begin{aligned} INF(v_i) = NTI(v_i) + NAI(v_i). \end{aligned}$$

For communities whose $m_{C_i}>0$, the *INF* value of nodes in this community will be calculated and the node with the maximum *INF* value will be selected as the seed node. To reduce the propagation overlap between seed nodes selected from the same community, the node will be removed from the network when it is selected as a seed node and the influence of its in-neighbors should be weakened. Suppose that node $v_j$ is a in-neighbour of node $v_i$, the topology and attribute influence of $v_j$ will be reduced if node $v_i$ is selected as the seed node. The updated topology influence $TI_{out}^{'}(v_j)$ and attribute influence $AI_{out}^{'}(v_j)$ can be calculated as:

17 $$\begin{aligned} \left\{ \begin{aligned} TI_{out}^{'}(v_j)&= TI_{out}(v_j)-1 \\ AI_{out}^{'}(v_j)&= AI_{out}(v_j)- \overline{s_a}(v_j,v_i). \end{aligned} \right. \end{aligned}$$

Then normalization topology and attribute influence of $v_j$ can be updated by taking Eq. (17) into Eq. (15), respectively. Finally, $INF(v_j)$ is also updated by recalculating Eq. (16). The node with the maximum *INF* will be selected as the seed node in each iteration. The proposed seed nodes selection method can be summarized as Algorithm 1.

#### Complexity analysis

We also analyze the time complexity of our proposed algorithm. Firstly, if DAGC method iterates *t* times, the time complexity of DAGC community detection is $O(N^2dt+ndt^2)$ where *N* is the number of nodes, *d* is the number of attributes and *n* is the number of nonzero entries of the adjacency matrix *A*^45^. Secondly, influence values for nodes in communities whose $m_{C_i}>0$ will be calculated in the seed nodes selection phase (as described in the 3th to 9th rows of Algorithm 1), which have a $O(l\cdot m_{C_i}\cdot |C_i|)$ complexity. Since $|C_i|$ can be approximated as the average value $\frac{N}{l}$ and $m_{C_i}$ is a constant, $O(l\cdot m_{C_i}\cdot |C_i|)\approx O(N)$. The complexity for recalculating influence of the selected node’s in-neighbors (as described in the 12th row of Algorithm 1) is $O(l\cdot m_{C_i} *N_{in}(v_i^*))$. Since $N_{in}(v_i^*)\ll |C_i|$, $O(l\cdot m_{C_i} *N_{in}(v_i^*))\ll O(N)$, the complexity of the seed nodes selection method is *O*(*N*). Overall, the total complexity of our proposed influence maximization algorithm is $O(N^2dt+ndt^2+N)$.

## Results

### Data description

We evaluate the performance of the proposed algorithm on five real world datasets and a large-scale synthetic dataset. Details of these datasets are described in Table 1. Five real world datasets including Pubmed, Cora, Cornell, Texas and Washington. The Pubmed dataset consists 19,717 scientific publications from PubMed database pertaining to diabetes classified into one of three classes. Its citation network consists 44,338 links. Each publication in the dataset is described by a TF/IDF weighted word vector from a dictionary which consists of 500 unique words. The Cora dataset consists 2708 scientific publications and 5429 links. Each publication in the dataset is described by a 0/1-valued word vector indicating the absence/presence of the corresponding word from the dictionary. The dictionary consists of 1433 unique words. The Cornell, Texas and Washington datasets are gathered from three different universities. Each line of these datasets contains two webpage IDs. The first entry is the ID of the webpage being cited and the second ID stands for the webpage which contains the citation. The synthetic large dataset named ‘Synthetic’ is constructed with 105,000 nodes and 830,159 edges. To generate our synthetic dataset, the function $random\_partition\_graph()$ in the networkx package of Python is used. More specifically, the number of community is set as 3 and the size of community is set as $[3\times 10^4, 3.5\times 10^4, 4\times 10^4]$. Nodes in the same community are connected with probability $2.5\times 10^{-4}$ and nodes of different communities are connected with probability $1\times 10^{-4}$. The attribute of each node is a vector of size 100. Initially, each bit of the vector is randomly assigned 0 or 1. When all neighbors of a node have attributes, the attribute of this node is rounding the average attribute value of its neighbors.

**Table 1:** Details of six datasets used in this paper.

Networks	Nodes	Edges	Communities	Attributes
Pubmed	19,717	44,338	3	500
Cora	2708	5429	7	1433
Cornell	195	304	5	1703
Texas	187	328	5	1703
Washington	230	446	5	1703
Synthetic	105000	830159	3	100

### Performance metrics

Two critical metrics are employed to evaluate the performance of our proposed algorithm in this paper:

- *Influence spread* $\sigma (S)$: for a given seed set *S*, the number of expected active nodes when the diffusion on the propagation model comes to steady state is denoted as $\phi (S)$. In the following experiments, $\phi (S)$ is the average value of 1000 times Monte-Carlo simulations. To facilitate observations on datasets of different scales, influence spread is defined as the ratio between $\phi (S)$ and the total number of nodes in the dataset:
  18 $$\begin{aligned} \sigma (S)=\frac{\phi (S)}{N}. \end{aligned}$$
  Influence spread is used to evaluate the effectiveness of an influence maximization algorithm. Higher $\sigma (S)$ value indicates that the algorithm is more effective.
- *Running time*: running time is defined as the time for selecting *m* seed nodes. In the previous community-based influence maximization study^38^, only the time of seed nodes selection phase is considered. To analyze the running time of the whole influence maximization algorithm in more detail, we report the running time of community detection, attribute similarity calculation (or K-Shell calculation for HGD and NCVoteRank) and seed nodes selection respectively, as shown in Table . The running time is measured in seconds.
- *Speedup*: the speedup is measured for influence spread of the proposed method over baseline methods with $m=30$, 40 and 50 seed nodes. The speedup^55^ is computed as:
  19 $$\begin{aligned} \text {speedup}=((A-B)/A)\times 100, \end{aligned}$$
  where *A* and *B* are the influence spread of two compared methods. For example, if the influence spread of Ours and K-Shell methods are 0.4475 and 0.2328, respectively, the speedup of Ours compared to K-Shell is calculated as: $\text {speedup}_{Ours\rightarrow K-Shell}=((0.4475-0.2328)\div 0.4475\times 100)=47.98$. Similarly, the speedup of K-Shell compared to ours is calculated as: $\text {speedup}_{K-Shell\rightarrow Ours}=((0.2328-0.4475)\div 0.2328\times 100)=-92.23$.

### Experimental results

**Figure 1:**
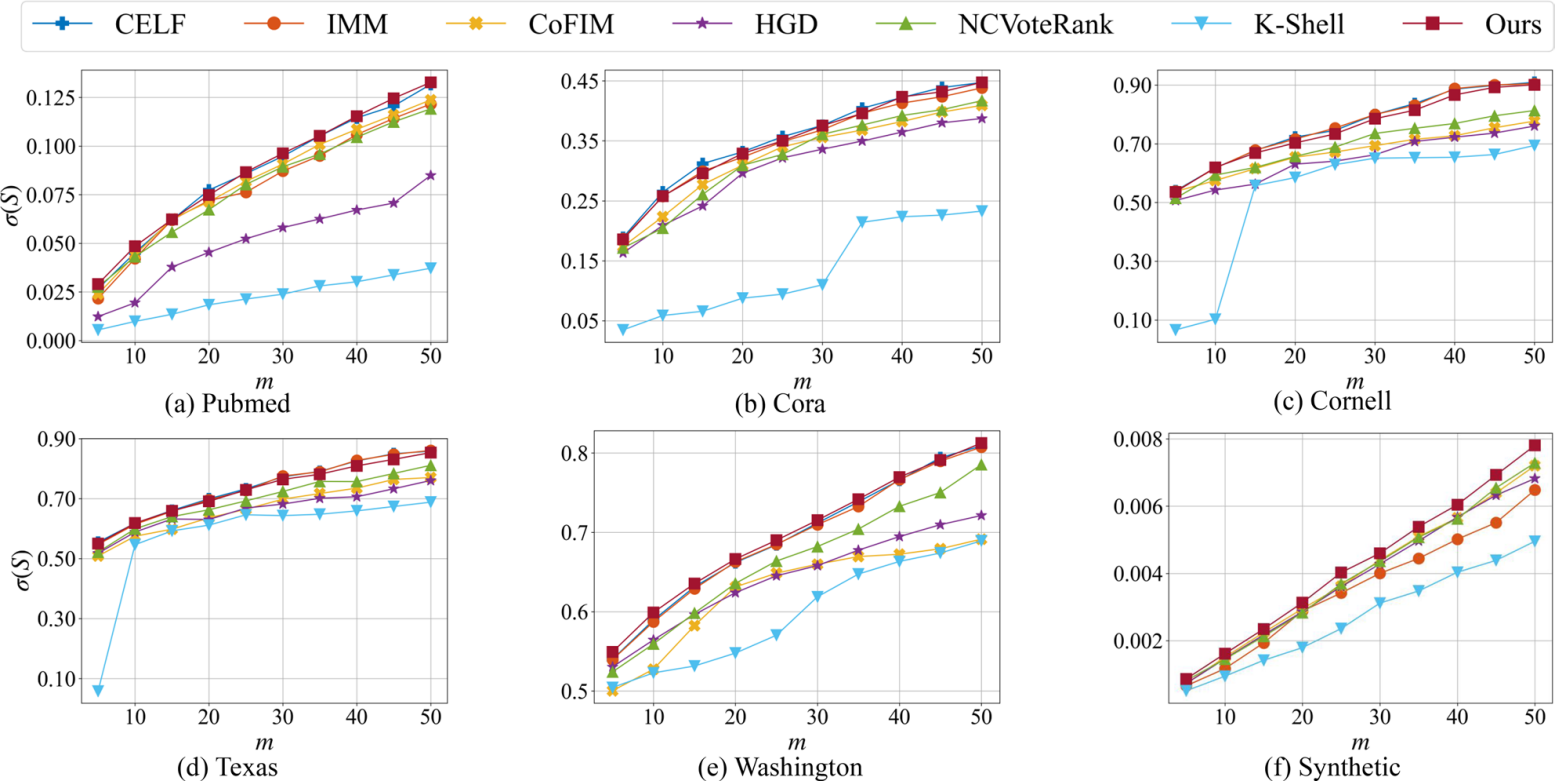
The influence spread $\sigma (S)$ of different algorithms on six datasets with different number of initial spreaders *m* under the proposed LTPlus model. The active threshold of each node is randomly sampled.

Based on the above networks, benchmark algorithms including CELF^6^, IMM^50^, CoFIM^38^, HGD^34^, NCVoteRank^32^, K-Shell^9^ are used to compare with our proposed method. To evaluate the effectiveness of our proposed method, we compare the influence spread $\sigma (S)$ of different algorithms under different initial numbers of seed nodes *m* on LTPlus model with random sampling the active threshold of each node. Results on six datasets are shown in Fig. 1, where x-axis represents the number of seed nodes *m* and y-axis represents the influence spread $\sigma (S)$. From the results, we can see that our method outperforms community-based method (CoFIM) and heuristic-based methods (HGD, NCVoteRank K-Shell) on all datasets. Besides, our proposed method surpasses CELF on Pubmed dataset in some scenarios. CELF and IMM have similar performance in influence spread on six datasets. On the four small datasets(Fig. 1b–e), our method has similar performance with CELF and IMM which have theoretical guarantees. However, CELF can not be executed on the Synthetic dataset since its running time is intolerable. Methods with no theoretical guarantees may perform well on some datasets, but perform poorly on other datasets. For example, NCVoteRank and CoFIM perform well on Pubmed and Synthetic but poorly on Washington. Since both topology and attribute influence are considered in the seed nodes selection process of Ours, our method is more stable than other methods without theoretical guarantees. Overall, from the influence spread results on six datasets, our proposed algorithm shows its effectiveness and robustness in finding influential seed nodes and achieving influence maximization.

**Figure 2:**
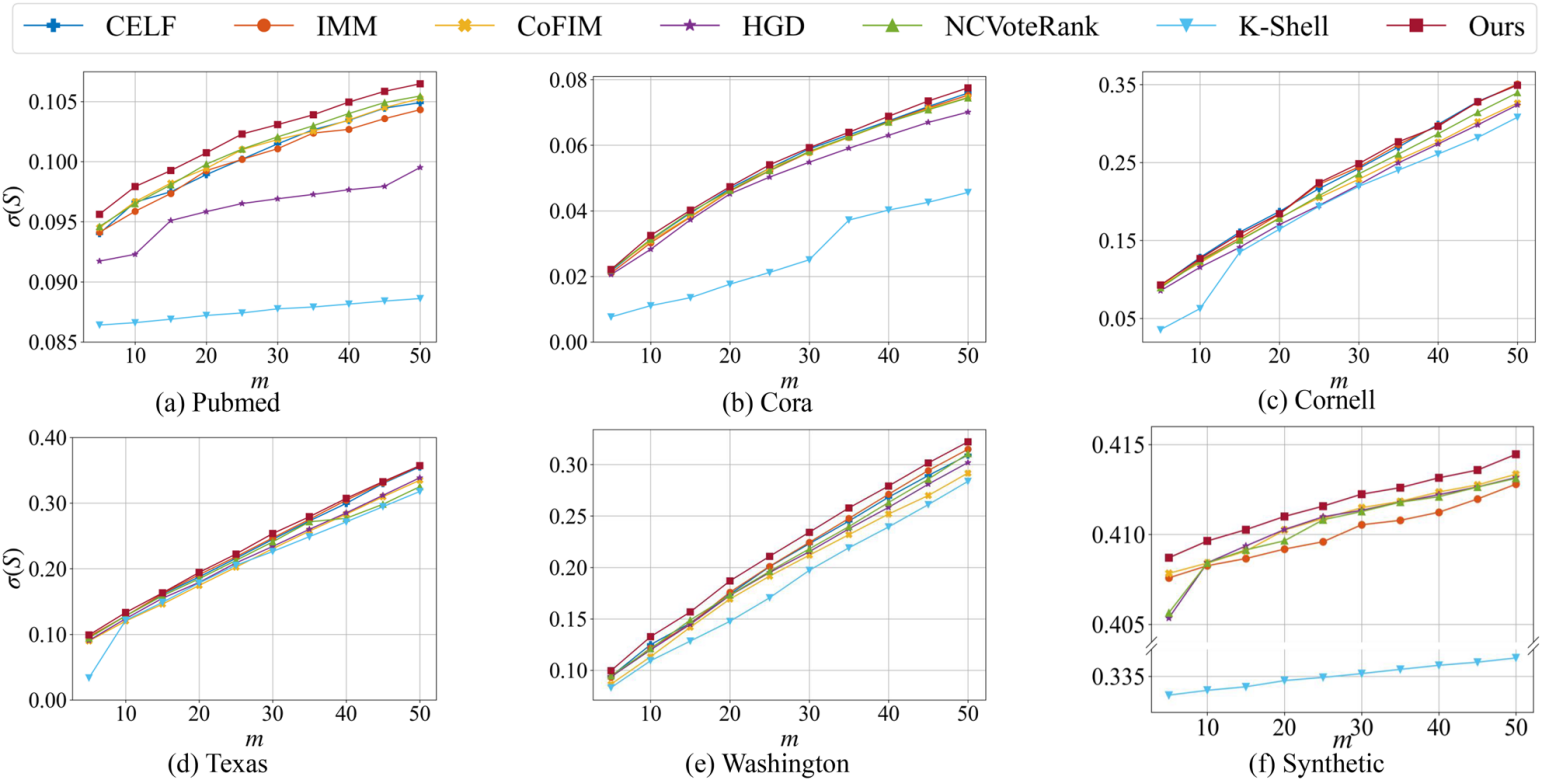
The influence spread $\sigma (S)$ of different algorithms on six datasets with different number of initial spreaders *m* under the IC model.

Since Independent Cascade (IC) model is also a classic propagation model, experiments are carried out on the IC model to evaluate the performance of the proposed method. In the IC model, a uniform probability *p* is assigned to each edge of the graph. A node $v_i$ has a chance of *p* to activate its out-neighbors. The probability *p* in our experiments is set as 0.1 by following the previous study^5^ and the number of seeds *m* ranges from 5 to 50. From Fig. 2, we can see that our proposed method still has a good performance in most cases. In addition, our node selection method does not depend on the propagation model, we do not need to re-select seeds when the propagation model changes. This proves the universality of our method.

The speedup experiments based on the LTPlus and the IC model are shown in Tables 3 and 4, respectively. Three different number of seeds 30, 40 and 50 are taken for experiments. Table 3 reveals that the proposed method has positive speedup than CoFIM, HGD, NCVoteRank and K-Shell on all datasets. Besides, the proposed method has positive speedup than CELF and IMM on Pubmed and Washington datasets. Although the proposed method has negative speedup than CELF and IMM on Cornell and Texas datasets, the absolute value of the speedup is very small, which means the difference of influence spread between these two methods is small. In Table 4, the proposed method has positive speedup than baseline methods in almost all datasets. The experimental results show the effectiveness of our proposed method.

In the seed nodes selection phase, we propose to recalculate the current influence of seed nodes’ in-neighbors (as shown in the 12th row of Algorithm 1) to reduce the propagation overlap between seed nodes selected from the same community. To verify the effectiveness of this step, we compare the influence spread of our proposed algorithm with/without recalulating *INF* of seed node’s in-neighbors, respectively. As shown in Table 2, the first row of each dataset is the influence spread of Ours method on the LTPLus model, and the second row of each dataset is the influence spread of our proposed method without recalculating *INF* of seed nodes’ in-neighbors in seed nodes selection phase, that is, without the 12th row in Algorithm 1. Compared to the method without recalculating *INF* in seed nodes selection phase, the influence spread of Ours method has an improvement to some extent. Especially in Washington network when $m=5$, the value of the first row is significantly higher than the second row. This may be due to that nodes in the network are concentrated in the same community and the number of initial seed nodes is small. Most seed nodes are selected from the same community and they may connect with each other. Seed nodes have a large number of common neighbors which eventually lead to a small influence spread. Therefore, it is necessary to recalculate the influence of seed nodes’ in-neighbors in the seed nodes selection process.

**Table 2:** Ablation experiments that analyze the impact of recalculating the *INF* of seed nodes’ in-neighbors.

Datasets	*m*									
	5	10	15	20	25	30	35	40	45	50
Pubmed	**0.0331**	**0.0525**	**0.0663**	**0.0788**	**0.0906**	**0.1003**	**0.1093**	**0.1194**	**0.1286**	**0.1368**
	0.0290	0.0491	0.0612	0.0752	0.0852	0.0957	0.1064	0.1157	0.1258	0.1343
Cora	**0.1756**	**0.2376**	**0.2760**	**0.3150**	**0.3300**	**0.3554**	**0.3759**	**0.4034**	**0.4119**	**0.4276**
	0.1635	0.2359	0.2635	0.2917	0.3175	0.3341	0.3573	0.3925	0.4042	0.4169
Cornell	**0.5358**	**0.6195**	**0.6688**	**0.7027**	**0.7335**	**0.7852**	**0.8144**	**0.8663**	**0.8921**	**0.9008**
	0.5269	0.6125	0.6593	0.6862	0.7174	0.7490	0.7915	0.8329	0.8638	0.8859
Texas	**0.5470**	**0.6155**	**0.6561**	**0.6879**	**0.7262**	**0.7613**	**0.7782**	**0.8061**	**0.8279**	**0.8504**
	**0.5470**	0.5804	0.6387	0.6607	0.6753	0.6921	0.7199	0.7553	0.7849	0.7984
Washington	**0.5492**	**0.5990**	**0.6354**	**0.6664**	**0.6901**	**0.7153**	**0.7417**	**0.7696**	**0.7902**	**0.8125**
	0.1133	0.5769	0.6111	0.6342	0.6631	0.6858	0.7015	0.7302	0.7670	0.7776
Synthetic	**0.0009**	**0.0016**	**0.0023**	**0.0031**	**0.0040**	**0.0046**	**0.0054**	**0.0060**	**0.0069**	**0.0078**
	**0.0009**	**0.0016**	0.0021	0.0029	0.0037	0.0043	0.0052	0.0058	0.0066	0.0074

The propagation is simulated on the LTPlus model and the number of seed nodes *m* changes from 5 to 50. The first and second row of each dataset is the influence spread of our method with/without recalulating *INF* of seed node’s in-neighbors, respectively.

Significant values are in bold.

**Table 3:** Speedup % (in terms of influence spread) for Ours versus other baseline methods on six datasets. The propagation is simulated on the LTPlus model.

Datasets	Seed size	Methods											
		Ours	CELF	Ours	IMM	Ours	CoFIM	Ours	HGD	Ours	NCVoteRank	Ours	K-Shell
Pubmed	30	1.59	−1.62	9.48	−10.48	5.75	−6.10	39.65	−65.71	7.16	−7.71	75.22	−303.63
	40	0.75	−0.75	8.21	−8.94	5.84	−6.20	41.82	−71.89	9.38	−10.35	73.81	−281.80
	50	0.80	−0.81	8.42	−9.19	6.79	−7.28	36.04	−56.34	10.24	−11.40	71.99	−257.05
Cora	30	−0.12	0.12	1.85	−1.89	5.24	−5.53	10.46	−11.68	3.84	−3.99	70.73	−241.65
	40	0.20	−0.20	2.45	−2.51	9.69	−10.74	13.86	−16.09	7.33	−7.91	47.22	−89.46
	50	0.02	−0.02	2.06	−2.10	8.58	−9.38	13.45	−15.54	6.82	−7.32	47.98	−92.23
Cornell	30	−1.78	1.75	−1.81	1.78	11.67	−13.21	15.63	−18.53	6.38	−6.82	17.11	−20.64
	40	−2.28	2.23	−2.50	2.44	16.07	−19.14	16.62	−19.93	11.24	−12.67	24.51	−32.47
	50	−0.90	0.89	−0.41	0.41	13.74	−15.93	15.60	−18.48	9.74	−10.79	22.94	−29.78
Texas	30	−1.11	1.09	−1.45	1.43	8.65	−9.47	10.79	−12.09	5.31	−5.60	15.82	−18.79
	40	−2.06	2.01	−2.27	2.22	9.17	−10.09	12.73	−14.59	6.48	−6.93	18.54	−22.76
	50	−0.58	0.57	−0.84	0.83	9.73	−10.78	10.89	−12.22	4.97	−5.23	19.36	−24.00
Washington	30	0.49	−0.49	0.79	−0.79	7.76	−8.41	7.99	−8.69	4.64	−4.86	13.49	−15.60
	40	0.50	−0.50	0.41	−0.41	12.62	−14.45	9.74	−10.79	4.78	−5.02	13.80	−16.01
	50	0.44	−0.44	0.62	−0.62	14.90	−17.52	11.23	−12.65	3.33	−3.44	15.16	−17.87
Synthetic	30	–	–	12.98	−14.91	4.47	−4.68	6.98	−7.50	5.25	−5.54	32.24	−47.57
	40	–	–	16.97	−20.45	6.51	−6.97	6.10	−6.49	6.96	−7.48	33.25	−49.81
	50	−	−	16.97	−20.44	7.77	−8.42	12.64	−14.46	6.65	−7.12	36.53	−57.55

**Table 4:** Speedup % (in terms of influence spread) for ours versus other baseline methods on six datasets.

Datasets	Seed size	Methods											
		Ours	CELF	Ours	IMM	Ours	CoFIM	Ours	HGD	Ours	NCVoteRank	Ours	K-Shell
Pubmed	30	1.55	−1.58	1.94	−1.98	1.21	−1.22	5.98	−6.36	0.99	−1.00	14.86	−17.45
	40	1.45	−1.47	2.17	−2.21	1.41	−1.43	6.95	−7.47	0.91	−0.91	16.01	−19.06
	50	1.47	−1.50	2.03	−2.07	1.16	−1.17	6.53	−6.99	0.96	−0.97	16.77	−20.16
Cora	30	0.64	−0.64	2.32	−2.38	2.44	−2.50	7.46	−8.06	1.84	−1.87	57.67	−136.25
	40	2.01	−2.05	2.39	−2.45	2.70	−2.77	8.42	−9.19	2.61	−2.68	41.55	−71.10
	50	2.04	−2.08	2.89	−2.97	3.75	−3.90	9.53	−10.53	3.93	−4.09	41.16	−69.96
Cornell	30	2.49	−2.55	1.74	−1.77	8.06	−8.76	10.89	−12.22	5.27	−5.56	11.74	−13.30
	40	−0.87	0.86	−0.38	0.38	6.83	−7.34	7.62	−8.25	3.21	−3.32	12.00	−13.63
	50	−0.26	0.26	−0.46	0.46	6.58	−7.04	7.23	−7.80	2.75	−2.83	11.78	−13.35
Texas	30	3.51	−3.63	2.60	−2.67	9.09	−9.99	7.75	−8.40	4.99	−5.25	10.78	−12.08
	40	2.48	−2.55	0.92	−0.93	7.88	−8.56	7.21	−7.77	9.88	−10.96	11.67	−13.22
	50	0.53	−0.54	0.06	−0.06	6.21	−6.63	5.18	−5.46	8.98	−9.86	11.01	−12.37
Washington	30	4.76	−5.00	4.21	−4.39	9.52	−10.52	8.07	−8.77	6.92	−7.44	15.82	−18.79
	40	3.89	−4.05	2.86	−2.94	9.75	−10.81	7.44	−8.04	5.72	−6.07	14.26	−16.63
	50	3.99	−4.16	2.25	−2.30	9.44	−10.42	6.25	−6.67	3.61	−3.74	11.92	−13.54
Synthetic	30	–	–	0.41	−0.41	0.18	−0.18	0.22	−0.22	0.23	−0.23	18.70	−23.00
	40	–	–	0.46	−0.47	0.19	−0.19	0.23	−0.23	0.25	−0.25	18.77	−23.10
	50	–	–	0.40	−0.40	0.27	−0.27	0.31	−0.31	0.32	−0.32	18.92	−23.34

The propagation is simulated on the IC model.

Time efficiency is a key indicator that many researchers concern about. Therefore, the running time of our proposed algorithm and baselines algorithms are analyzed in stages. Experiments are carried out on a computer with 2.30 GHz Intel i7-10875H CPU and 32GB memory. Table  shows the running time of various algorithms on six datasets. Here the running time of seed nodes selection is the time of selecting 25 seed nodes. As can be seen from this table, the time efficiency of our proposed method is very competitive in seed nodes selection phase. Although CELF has a good performance in influence spread, its running time is too long. IMM shows high time efficiency in all datasets. However, both CELF and IMM select seeds depend on the propagation model. They should reselect seeds when the propagation model changed. CoFIM has a relative high time efficiency in the seed nodes selection process in large-scale datasets. The running time of K-Shell is low, but its influence spread is unsatisfactory. HGD and NCVoteRank show high time efficiency in some datasets but sometimes it is inefficient and their influence spread performance is also not stable.

**Table 5:** Running time (in seconds) for different algorithms on six datasets.

Phase	Datasets	CELF	IMM	CoFIM	HGD	NCVoteRank	K-Shell	Ours
Community detection	Pubemd	–	–	14.43	–	–	–	69.43
	Cora	–	–	1.57	–	–	–	28.83
	Cornell	–	–	0.17	–	–	–	2.28
	Texas	–	–	0.18	–	–	–	1.67
	Washington	–	–	0.20	–	–	–	2.26
	Synthetic	–	–	1616.40	–	–	–	3374.65
Attribute similarity calculation (or K-Shell calculation*)	Pubemd	4.74	4.74	–	6.44*	6.44*	6.44*	4.74
	Cora	0.47	0.47	–	0.88*	0.88*	0.88*	0.47
	Cornell	0.05	0.05	–	0.16*	0.16*	0.16*	0.05
	Texas	0.05	0.05	–	0.07*	0.07*	0.07*	0.05
	Washington	0.05	0.05	–	0.10*	0.10*	0.10*	0.05
	Synthetic	–	29.43	–	117.49*	117.49*	117.49*	29.43
Seed nodes selection	Pubemd	101,232.05	0.38	0.31	0.36	0.41	–	0.56
	Cora	1977.51	0.05	0.16	0.22	0.04	–	0.16
	Cornell	14.33	0.01	0.08	0.11	0.02	–	0.02
	Texas	14.65	0.01	0.08	0.09	0.03	–	0.02
	Washington	24.25	0.01	0.11	0.17	0.03	–	0.02
	Synthetic	–	2.53	0.56	2.15	13.65	–	2.98

The number of seed nodes *m* is 25. Symbol ‘–’ indicates that the corresponding cell has no value.

Besides, except for the time of seed nodes selection phase, the community detection time of Ours and CoFIM is also analyzed. Compared with CoFIM, the graph-embedding based community detection method used in Ours requires more time to find proper communities. Although the community detection phase seems to be time-consuming, it only needs to be carried out once for each dataset, no matter how many groups of experiments are carried out on one dataset. The time of calculation attribute similarities in CELF and Ours under the LTPlus model is reported. Similarly, the time of calculation K-Shell values in HGD and NCVoteRank is also reported. It should be noted that attribute similarities and K-Shell values are computed and saved in advance for the convenience of multiple experiments. That is, they are only executed one time for each dataset.

## Discussion

In summary, we propose an extension of LT information propagation model, named LTPlus, that considers topologies and attributes of nodes in propagation simulations. This model is more suitable than previous information propagation models in attributed networks. In addition, we propose a novel community-based method to identify a set of vital nodes to achieve influence maximization in attributed networks. To the best of our knowledge, the proposed method makes the first effort to combine influence maximization with the graph-embedding community detection method. Compared with well-known state-of-the-art methods, empirical analyses on five real world networks and a large scale synthetic network under the LTPlus model suggest that our proposed method always performs very competitively, as shown in Fig. 1. Experimental results in Fig. 2 show the universality of our proposed method under the IC model. We believe our work can bring a little light into studies of the influence maximization problem in the future. For example, the graph-embedding community detection method can be further improved for directed attributed networks. In addition, an end-to-end method considering the property of propagation models can be further explored in the future work.

## Supplementary Information

Supplementary Information.

## Data Availability

All relevant real world datasets can be downloaded from https://github.com/yingwang926/attributed_datasets.
