# Supplementary material for: Identifying vital nodes for influence maximization in attributed networks
Source: Sci Rep. 2022 Dec 31;12:22630. doi: 10.1038/s41598-022-27145-3 (PMC9805466; doi:10.1038/s41598-022-27145-3)
Supplement: Supplementary file 1 — Supplementary Information. [file 41598_2022_27145_MOESM1_ESM.zip › appendix/appendix.pdf]

Appendix A. Monotonicity and submodularity of $\phi(S)$ under the LTPlus model

As shown in Eq.(3) and Eq.(5), $TI_{in}(v_j, v_i) \in (0,1)$ and $AI_{in}(v_j, v_i) \in (0,1)$. Since $\alpha_1, \alpha_2 \in (0,1)$ and $\alpha_1 + \alpha_2 = 1$, the range of b_{v_j,v_i} in Eq.(6) is in (0,1) and $\sum_{v_j \in N_{in}(v_i)} b_{v_j,v_i} \leq 1$. Thus, we can follow the general methodology in [5] to prove that $\phi(S)$ is monotone and submodular under the LTPlus model.

For the given directed and attributed network $G = (V, E, X)$, where V is the node set, E is the edge set and X is the node attribute matrix, we can construct a random live-edge graph G_l which is an equivalent random process to the LTPlus model. More specifically, for each node $v_i \in V$, $(v_j, v_i) \in E$ is selected exclusively as the single live edge with probability b_{v_j,v_i} among all incoming edges of v_i, or no edge is selected as a live edge with probability $1 - \sum_{v_j \in N_{in}(v_i)} b_{v_j,v_i}$.

First, we discuss the probability that a node can be activated in the activation process of LTPlus model. Given a seed node set S, S_t is defined as the set of active nodes at the end of iteration t for $t = 0, 1, 2 \ldots$, and $S_0 = S$. Here we consider a node v_i which has not been activated by the end of iteration t, namely $v_i \notin S_t$. Thus, the probability v_i becomes active in iteration $t+1$ equals to the chance that influence weights in $S_t \setminus S_{t-1}$ push it over its active threshold. This probability is $\frac{\sum_{v_j \in S_t \setminus S_{t-1}} b_{v_j,v_i}}{1 - \sum_{v_j \in S_{t-1}} b_{v_j,v_i}}$.

Then, we consider the above discussed probability when using the random live-edge graph. If node v_i is inactive by the end of iteration t, it means that v_i has no live edge from S_{t-1} in the random live-edge graph. Then, the probability that v_i is activated by the end of iteration $t+1$ is equal to the chance that its live edge comes from $S_t \setminus S_{t-1}$. By the definition of the random live-edge graph, this probability is $\frac{\sum_{v_j \in S_t \setminus S_{t-1}} b_{v_j,v_i}}{1 - \sum_{v_j \in S_{t-1}} b_{v_j,v_i}}$ which is the same as the LTPlus model. Thus, the random live-edge graph model produces the same distribution over active sets as the LTPlus model.

With the equivalence of the live-edge graph and the LTPlus model, we just need to show the monotonicity and submodularity of $\phi(S)$ in the random live-edge graph. Let ζ denotes the set of all possible live-edge graphs of G, and the probability that a random live-edge graph G_l selected from ζ is $Pr(G_l)$. Given the seed node set S, the active node set in G_l is set as $A_{G_l}(S)$.

Therefore, the expected number of active nodes is

$$\phi(S) = \sum_{G_l \in \zeta} Pr(G_l)|A_{G_l}(S)| \tag{A.1}$$

The number of active nodes is a monotone increasing function of the seed set. Thus, $|A_{G_l}(S)|$ is a monotone increasing function. Since the linear combination of monotone (resp. submodular) functions with non-negative coefficients is also monotone (resp. submodular). The monotonicity of $\phi(S)$ is proved. To prove the submodularity, for any two subsets $S \subseteq T \subseteq V$ and a node $v \in V \setminus T$, we need to prove that $A_{G_l}(T \cup v) \setminus A_{G_l}(T) \subseteq A_{G_l}(S \cup v) \setminus A_{G_l}(S)$. For node $u \in A_{G_l}(T \cup v) \setminus A_{G_l}(T)$, u is reachable from $T \cup v$ but not reachable from T in G_l. That is, u is reachable from v. Since $S \subseteq T$, u is not reachable from S but can be reachable from $S \cup v$. That is, $u \in A_{G_l}(S \cup v) \setminus A_{G_l}(S)$. Thus, $|A_{G_l}(S)|$ is submodular in all G_l, which means $\phi(S)$ is submodular under the LTPlus model.
